# Arachidonic Acid in Follicular Fluid of PCOS Induces Oxidative Stress in a Human Ovarian Granulosa Tumor Cell Line (KGN) and Upregulates GDF15 Expression as a Response

**DOI:** 10.3389/fendo.2022.865748

**Published:** 2022-05-11

**Authors:** Yalan Ma, Lianwen Zheng, Yeling Wang, Yiyin Gao, Ying Xu

**Affiliations:** ^1^ Reproductive Medical Center, Department of Obstetrics and Gynecology, The Second Hospital of Jilin University, Changchun, China; ^2^ Cardiovascular Medicine Department, The First Hospital of Jilin University, Changchun, China

**Keywords:** PCOS, arachidonic acid, oxidative stress (OS), human ovarian granulosa tumor cell line (KGN), GDF15, response

## Abstract

Polycystic ovarian ovary syndrome (PCOS) is the main cause of ovulatory infertility and a common reproductive endocrine disease of women in reproductive age. In addition, nearly half of PCOS patients are associated with obesity, and their total free fatty acids tend to increase. Arachidonic acid (AA) is a polyunsaturated fatty acid. Oxidation products of AA reacting with various enzymes[cyclooxygenases (COX), lipoxygenases (LOX), cytochrome P450s (CYP)] can change cellular mitochondrial distribution and calcium ion concentration, and increase reactive oxygen species (ROS) production. In this study, we analyzed the follicular fluid fatty acids and found higher levels of C20:4n6 (AA) in PCOS patients than in normal control subjects. Also, to determine whether AA induces oxidative stress (OS) in the human ovarian granulosa tumor cell line (KGN) and affects its function, we treated KGN cells with or without reduced glutathione (GSH) and then stimulated them with AA. The results showed that AA significantly reduced the total antioxidant capacity (TAC) and activity of antioxidant enzymes and increased the malondialdehyde (MDA), ROS and superoxide anion(O_2-_)levels in KGN cells. In addition, AA was also found to impair the secretory and mitochondrial functions of KGN cells and induce their apoptosis. We further investigated the downstream genes affected by AA in KGN cells and its mechanism of action. We found that AA upregulated the expression of growth differentiation factor 15 (GDF15), which had a protective effect on inflammation and tissue damage. Therefore, we investigated whether AA-induced OS in KGN cells upregulates GDF15 expression as an OS response.Through silencing of GDF15 and supplementation with recombinant GDF15 (rGDF15), we found that GDF15, expressed as an OS response, protected KGN cells against AA-induced OS effects, such as impairment of secretory and mitochondrial functions and apoptosis. Therefore, this study suggested that AA might induce OS in KGN cells and upregulate the expression of GDF15 as a response to OS.

## Introduction

Polycystic ovary syndrome (PCOS) is one of the most common reproductive endocrine diseases, with a prevalence rate of 5-10% (1). Its clinical manifestations vary, and include amenorrhea, hirsutism, obesity, hyperinsulinemia, hyperandrogenemia, presence of polycystic ovary in ultrasound examination, *etc* (2). Patients with PCOS produce more oocytes after stimulation of ovulation than non-PCOS patients. However, the growth and fertilization rate of oocytes and embryo rate are reduced, which is consistent with other studies (3, 4). Follicular fluid provides a living environment for the development and maturity of oocytes. Therefore, changes in the composition of follicular fluid can affect the quality of oocytes, including the maturation, fertilization, cleavage and early embryo formation of oocytes (5). In addition, the follicular fluid contains a large amount of polyunsaturated fatty acids (6). In our study, it was found that the AA in follicular fluid of PCOS patients after hyperovulation was higher than that of the control group. AA is one of the most abundant, active and widely distributed polyunsaturated essential fatty acids in the human body (7, 8). Enzymatic oxidation products of AA reactions, catalyzed by various enzymes (COX, LOX, CYP, *etc.*) (9–11), are involved in almost the entire reproductive process, including oocyte maturation, ovulation, implantation, delivery, *etc.* In addition, AA plays an important role in inflammation and is closely related to OS (12). Therefore, the study of the relationship between the two can lead to a better understanding of the pathogenic mechanism of PCOS, which is of great importance to the research on the etiology and treatment of the change *in vitro* fertilization (IVF)/intracytoplasmic sperm injection (ICSI) assisted pregnancy of PCOS patients. Investigating the association between AA-induced OS and the expression of GDF15 in KGN cells established a theoretical basis for the development of a treatment strategy to improve pregnancy outcome of PCOS patients.

## Materials and Methods

### Participants

Sixteen women with PCOS were selected and enrolled in this study according to the revised Rotterdam consensus guidelines (13). Additionally, another 16 women with infertility due to simple tubal factors were also selected and enrolled in this study as controls. These patients had regular menstruation, normal ovarian function, and no androgenic clinical and biochemical characteristics. The patients in the PCOS group were divided into PCOS normal weight group (8 cases, 18.5 kg/m^2^ ≤BMI<24 kg/m^2^) and PCOS overweight group (8 cases, BMI ≥ 24 kg/m^2^), according to the BMI of Chinese people which was developed by the International Society for Life Sciences according to the body type of Chinese people (14). In the control group, subjects were divided into normal weight group (8 cases, 18.5 kg/m^2^ ≤ BMI<24 kg/m^2^) and overweight group (8 cases, BMI ≥ 24 kg/m^2^). Ovulation induction regimens in both groups were modified long protocols and all patients were between 25 and 35 years old. Patients with the following diseases were excluded from the study: (1) Patients with other conditions that cause ovulation dysfunction, including hyperprolactinemia, early-onset ovarian insufficiency, hypogonadotropin amenorrhea, and thyroid dysfunction. (2) Women with other causes of hyperandrogenism, such as congenital adrenal hyperplasia, androgen-secreting tumors, Cushing’s syndrome, *etc* (15). All patients signed informed consent for this study. The study was approved by the Ethics Committee of the Second Hospital of Jilin University (Jilin, China 2020 Review No. 123).

### Controlled Ovarian Hyperstimulation and Follicular Fluid Collection

All patients were enrolled in a modified long protocol. On the second day of menstruation, leprorelin acetate (GnRH-a, 3.75 mg/dose) was subcutaneously injected with 0.935 mg. After administering the injection for18-20 days, the drop regulation was adjusted according to the level of sex hormones and the results of the ultrasound examination. If it did not meet the criteria, another subcutaneous injection of 1.25 mg of leprorelin acetate was given. Gn was used when the down-regulation standard was reached (16). When there were 2 dominant follicles with average diameter ≥18 mm or 3 dominant follicles with average diameter ≥18 mm and gt, Gn was stopped at 17 mm. Then, 250 µg of recombinant human chorionic gonadotropin was injected on the same night, and ovum pick up (OPU) was performed 34-36 h later. Follicular fluid from 18-20 mm diameter follicles was collected during OPU(to prevent blood contamination), centrifuged immediately (800g*10 min) and supernatant was collected and placed in a 1.5 mL cryopreserved tube and stored at -80°C.

### Extraction, Measurement and Calculation of Fatty Acids in Follicular Fluid

#### Pre-Processing Method

##### (1) Sample Hydrolysis

Firstly, we weigh an appropriate amount of uniform sample, add about 100 mg of pyrogallic acid, add a few grains of zeolite, and then add 2 mL of 95% ethanol, and mix well. Secondly, we add 10 mL of hydrochloric acid solution and mix. The flask was placed in a water bath at 70°C to 80°C for hydrolysis for 40 min. Then, we shake the flask every 10 min to mix the particles adhering to the walls of the flask into the solution. After the hydrolysis was complete, the flask was removed and cooled to room temperature.

##### (2) Extraction of Fat

Firstly, we add 10 mL of 95% ethanol to the hydrolyzed sample, and mix well. Secondly, we transfer the hydrolyzate in the flask to a separatory funnel, rinse the flask and stopper with 50 mL of ether-petroleum ether mixture, and incorporate the rinse into the separatory funnel and cap it. Shake for 5min and let stand for 10min. The ether layer extract was collected into a 250 mL flask. Then, we repeat the extraction of the hydrolyzate 3 times according to the above steps, and finally rinse the separatory funnel with a mixture of ether and petroleum ether, and collect it into a flask with constant weight, put the flask on a water bath and evaporate to dryness and dry it in an oven at 100°C ± 5°C for 2 hours.

##### (3) Saponification of Fat and Fatty Acid Methylation

Firstly, in the fat extraction, we continue to add 2 mL of 2% sodium hydroxide methanol solution, water bath in a water bath at 85°C for 30 min and add 3 mL of 14% boron trifluoride methanol solution. Water bath at 85°C for 30 min. Secondly, after the water bath is completed, we wait for the temperature to drop to room temperature, add 1 mL of n-hexane to the centrifuge tube, shake and extract for 2 min, and let it stand for one hour to wait for stratification. Then, we take 100 μL of the supernatant, dilute to 1 mL with n-hexane and use 0.45 μm filter membrane to test on the machine. Finally, after hydrolyzing the sample, the fat was extracted and saponified, and the fatty acids were methyl esterified.

Then, the fatty acids were detected by gas chromatography-mass spectrometry (GC-MS). The contents of each fatty acid in the sample were calculated using the corresponding formula.

### Origin and Culture of the Human Ovarian Granulosa Tumor Cell Line (KGN)

The immortal human ovarian granulosa tumor cell line cell line (KGN) was used in this study. This cell line was derived from granulosa cells of a patient with ovarian cancer and immortalized. It has the ability to synthesize steroid hormones and the growth characteristics of granulosa cells, so it is often used to study the function, proliferation and hormone regulation of granulosa cells. The frozen KGN cells were quickly thawed in a 37°C water bath and quickly transferred to a centrifuge tube containing 10 mL of serum-free medium. After centrifugation at 1,200 rpm for 7 min, the precipitated cells were transferred to Dulbecco’s modified Eagle’s medium (DMEM) containing 10% fetal bovine serum and well mixed. The cells were cultured in a cell incubator at 37°C with 5% CO_2_ and the cell morphology and density were observed. When the cell confluence reached 80-90%, cells were detached by digestion with 0.05% trypsin containing EDTA and passaged.

### Treatment of KGN Cells

KGN cells were stimulated with different concentrations (0, 25, 50, 100 and 200 μM) of AA and the optimum concentration was selected for subsequent experiments. After treating KGN cells with different concentrations of AA, we examined whether these concentrations have different effects on mitochondrial function and cell viability. In addition, KGN cells were exposed to 50 μM AA for 12 h with or without GSH (5 mmol/L). KGN cells pretreated with or without buthionine sulfoximine (BSO) (10 μM)for 1h, and added with or without recombinant GDF15 (50ng/ml)(rGDF15; Abnova, Taiwan, China) for 4 h in advance, were also exposed to 50 μM AA for 12 h. KGN cells transfected with GDF15 small-interfering RNA (siRNA) for 6 h and then culture was continued by replacing the medium. Finally, KGN cells were also exposed to 50 μM AA for 12 h in the presence or absence of 20 μM Mito-TEMPO.

### Cell Viability

Briefly, the KGN cells were seeded into a 96-well plate and treated with AA at different concentration for 12 h. Then, 10 μL of Cell Counting Kit-8 (CCK-8; Beyotime Biotechnology, Shanghai, China) solution was added into each well and the plate was incubated at 37°C for 1 h. The optical density (OD) of each well was measured at 450 nm on a microplate reader(BioTek Instruments, Winooski, VT, USA).

### Measurement of ROS, O_2-_ and mitoSOX Levels

KGN cells were treated as described above, and the cells were incubated with 20 μM dichlorodihydrofluorescein diacetate (DCFH-DA; Beyotime Biotechnology) for 20 min, followed by 10 μM dihydroethidium (Beyotime Biotechnology) for 30 min or 5 μM MitoSOX Red mitochondrial superoxide indicator (Invitrogen, Carlsbad, CA, USA) for 10 min. Intracellular ROS, O_2-_ and mitoSOX levels were measured by flow cytometry.

### Measurement of Parameters Related to Pro-Oxidation and Anti-Oxidation

After subjecting the KGN cells to the appropriate treatment, their proteins were extracted. Then, the contents of malondialdehyde (MDA) and reduced glutathione (GSH) were measured using to the corresponding assay kit (Beyotime Biotechnology). In addition, the activities of superoxide dismutase (SOD), catalase (CAT), glutathione peroxidase (GSH-Px) and glutathione reductase (GR) were determined using to the corresponding assay kit (Beyotime Biotechnology).

### Measurement of Mitochondrial Membrane Potential and ATP Content

After the appropriate treatment, KGN cells were respectively incubated with JC-1 and tetramethylrhodamine ethyl ester (TMRE) fluorescent probes and hoechst staining using the corresponding mitochondrial membrane potential detection kit (Beyotime Biotechnology). Then, probes and dye were detected by flow cytometry and immunofluorescence microscopy. The ATP content was measured according to the instructions and the corresponding kit (Beyotime Biotechnology)

### Measurement of Levels of Hormones

After treatment, culture supernatants were collected. The estradiol (E_2_) and progesterone (P) levels were measured using the corresponding enzyme-linked immunosorbent assay (ELISA) kit (Cusabio Technology LLC, Houston, TX, USA).

### Cell Apoptosis Analysis

After the appropriate treatment, KGN cells were incubated with annexin V-fluorescein isothiocyanate (FITC) and propidium iodide (PI) according to the manufacturer’s instructions (Beyotime Biotechnology). Then, cell apoptosis rate was determined by flow cytometry. In addition, Caspase3 activity was determined using the corresponding assay kit (Beyotime Biotechnology).

### RNA Sequencing and Bioinformatics Analysis

Each group of 3 KGN cells RNA samples with RNA Integrity Number (RIN) ≥ 7 were sequenced by Sangon Biotech Co, Ltd. (Shanghai, China). We used the Gene Ontology (GO) database to functionally categorize the differentially expressed genes. The possible signaling pathways associated with these differentially expressed genes were identified using the Kyoto Encyclopedia of Genes and Genomes (KEGG) database.

### RNA Interference

After introducing the GDF15 siRNA into KGN cells using the Lipofectamine 3000 protocol cells were collected at 48 h with or without Mito-TEMPO.Primer sequences are listed in **Table 1**.

**Table 1:** The primer sequences for small-interfering RNAs (siRNAs) targeting GDF15.

siRNAs	Primer
siRNA-1(sense)	5’-CUCAGAGUUGCACUCCGAATT-3’
siRNA-1(antisense)	5’-UUCGGAGUGCAACUCUGAGTT-3’
siRNA-2(sense)	5’-CCGGAUACUCACGCCAGAATT-3’
siRNA-2(antisense)	5’-UUCUGGCGUGAGUAUCCGGTT-3’
siRNA-3(sense)	5’-GCUCCAGACCUAUGAUGACTT-3’
siRNA-3(antisense)	5’-GUCAUCAUAGGUCUGGAGCTT-3’
negative control(sense)	5’-UUCUCCGAACGUGUCACGUTT-3’
negative control(antisense)	5’-ACGUGACACGUUCGGAGAATT-3’

### Quantitative Real-Time Quantitative Polymerase Chain Reaction (qRT-PCR) Analysis

Total RNA was extracted from KGN cells using the appropriate RNA extraction kit (Beyotime Biotechnology) according to the manufacturer’s instructions. The extracted RNA was reverse transcribed using a reverse transcription kit. Polymerase chain reaction (PCR) was used to measure the expression of target genes, and quantitative real-time PCR (qRT-PCR) was used to determine the dynamic abundance of target genes. Relative mRNA expression of the following genes was analyzed by 2^−ΔCT^ method. Primer sequences are listed in **Table 2**.

**Table 2:** The primer sequences for CYP11A1, CYP19A1, STAR, HSD3B1, BAX, BCL2, Caspase3 and GAPDH.

Gene	Primer
CYP11A1(sense)	5’-CCCTGTTGGATGCAGTGTCT-3’
CYP11A1(antisense)	5’-TTGAGCACAGGGTACTTTA-3’
CYP19A1(sense)	5’-GGACCCCTCATCTCCCACG-3’
CYP19A1(antisense)	5’-CCCAAGTTTGCTGCCGAAT-3’
STAR(sense)	5’-CAGACTTCGGGAACATGCCT-3’
STAR(antisense)	5’-GGGACAGGACCTGGTTGATG-3’
HSD3B1(sense)	5’-AGCATCCGAGGACAGTTCTAC-3’
HSD3B1(antisense)	5’-AGGGCGGTCGATAGGTGTAA-3’
BAX(sense)	5’-ACGGCCTCCTCTCCTACTTT-3’
BAX(antisense)	5’-GCCTCAGCCCATCTTCTTC-3’
BCL-2(sense)	5’-GCCGGTTCAGGTACTCAGTC-3’
BCL-2(antisense)	5’-GCCGGTTCAGGTACTCAGTC-3’
Caspase3(sense)	5’-CTGGACTGTGGCATTGAGAC-3’
Caspase3(antisense)	5’-GCAAAGGGACTGGATGAACC-3’
GAPDH(sense)	5’-ATTTGGCTACAGCAACAGG-3’
GAPDH(antisense)	5’-TTGAGCACAGGGTACTTTATT-3’

Steroidogenic acute regulatory protein (STAR), cytochrome P450 family 11 subfamily a member 1 (CYP11A1), hydroxy-delta-5-steroid dehydrogenase, 3β-hydroxysteroid dehydrogenase(HSD3B1), cytochrome P450 family 17 subfamily a polypeptide 1 (CYP19A1), cytochrome P450 family 19 subfamily a member 1 (CYP19A1).

### Western Blot Analysis

After the appropriate treatment, the proteins from KGN cells were extracted and the protein concentration was determined using the bicinchoninic acid (BCA) Protein Assay Kit (Beyotime Biotechnology) according to the manufacturer’s instructions. Then, the samples were subjected to sodium dodecyl sulfate-polyacrylamide gel electrophoresis (SDS-PAGE) and then transferred to polyvinylidene fluoride (PVDF membranes. Antibodies against GDF15 (1:1,000; Cell Signaling Technology, Danvers, MA, USA), or β-actin (1:2,000; Proteintech Group, Rosemont, IL, USA) were incubated overnight with the membranes followed by incubation with the appropriate horseradish peroxidase (HRP)-conjugated secondary antibody. The signal was visualized using an enhanced chemiluminescence substrate kit.

### Statistical Analysis

All the results are presented as the means ± standard error of mean (SEM) and each experiment was performed in triplicate. Statistical analyses were performed with the GraphPad Prism8 software (GraphPad Software Inc., San Diego, CA, USA) and the SPSS v.17.0 software (IBM Corporation, Armonk, NY, USA). When a one-way analysis of variance (ANOVA) indicated significant differences among groups, a value of P < 0.05 was considered statistically significant.

## Results

### Analysis of IVF Cycle Parameters of the Study Population

The general characteristics of the patients are shown in **Table 3**. There were no significant differences between the groups in terms of individual characteristics and basic clinical data, including age, BMI, or serum FSH, E_2_, P on days 3–5 of the menstrual cycle. However, the serum testosterone level on days 3–5 of the menstrual cycle and duration of infertility was higher level in the PCOS patients than in the control subjects (P<0.01)(**Table 3**). Furthermore, the E_2_ and testosterone levels on the day of administration were significantly higher in PCOS patients than those in control subjects (P<0.05) (**Table 4**). In addition, embryo implantation rate was considerably lower in the PCOS patients than in the control subjects (P < 0.05) (**Table 4**).

**Table 3:** Clinical characteristics of the study participants between control and PCOS group $\bar{\text{X}}\pm \text{S}$ .

Variables	Control (n=16)	PCOS (n=16)	P-value
Age(y)	31.81 ± 3.94	31.88 ± 4.10	0.965
Duration of infertility(y)	3.06 ± 1.34	6.00 ± 3.95	<0.01*
BMI^a^ (Kg/m^2^)	23.04 ± 1.87	23.69 ± 1.75	0.313
Basal FSH^b^(U/L)	4.35 ± 2.30	5.16 ± 2.42	0.341
Basal LH^c^ (U/L)	4.21 ± 1.22	3.79 ± 2.10	0.502
Basal E2(pg/ml)	53.39 ± 19.12	48.88 ± 18.73	0.505
Basal Testosterone(ng/mL)	0.38 ± 0.15	0.65 ± 0.09	<0.01^*^
Total gonadotropin dose (IU)	2812.5 ± 978.26	2343.75 ± 812.64	0.151
LH level on the day of hCG administration(mIU/m L)	1.65 ± 0.30	1.73 ± 0.74	0.627
E2 level on the day of administration(pg/ml)	2394.28 ± 597.18	3909.52 ± 984.34	<0.01^*^
P level on the day of administration (ng/mL)	1.06 ± 0.56	1.03 ± 0.40	0.857
Testosterone level on the day of administration(ng/mL)	0.34 ± 0.12	0.82 ± 0.42	0.025^*^

^a^BMI, body mass index; ^b^FSH, Follicle- stimulating hormone; ^c^LH, Luteinizing Hormone; ^d^E2, estradiol.

**Table 4:** Laboratory and pregnancy outcomes of the study participants between control and PCOS group $\bar{\text{X}}\pm \text{S}$ .

Variables	Control (n=16)	PCOS (n=16)	P-value
Cycles(n)	16	16	—
Number of oocytes retrieved (n)	7.50 ± 1.32	8.00 ± 3.38	0.586
Number of mature oocytes (n)	6.75 ± 1.29	7.75 ± 3.26	0.262
Number of normal fertilized oocytes (n)	6.13 ± 1.01	6.50 ± 2.76	0.616
Fertilization rated^d^ (%)	81.67 (98/120)	81.25 (104/128)	0.933
Number of cleaved embryos Cleavage rate^e^ (%)	72.45 (71/98)	64.42 (67/104)	0.159
Number of available embryos	4.44 ± 2.42	4.19 ± 1.17	0.712
Number of good quality embryos	3.06 ± 1.69	3.13 ± 1.09	0.902
Good quality embryo rate^f^ (grade I and II) (%)	69.01 (49/71)	74.63 (50/67)	0.587
Embryo implantation rate^g^ (%)	50.00 (26/48)	28.57 (18/56)	0.039*
Cumulative clinical pregnancy rate^h^ (%)	58.30 (14/24)	39.29 (11/28)	0.275

Data are reported as mean ± standard deviation.

^d^Fertilization rate, number of fertilized oocytes/number of oocytes retrieved; ^e^Cleavage rate, number of cleaved embryo/number of fertilized oocytes; ^f^Good quality embryo rate, grade I and II/number of cleaved embryo ^g^Embryo implantation rate, Number of gestational sac/number of embryos transferred. ^h^Cumulative clinical pregnancy rate, presence of embryonic heartbeat visualized by ultrasound at 6 weeks after embryo transfer/number of transplantation cycles.

### Fatty Acid Composition in Follicular Fluid

**Figure 1:**
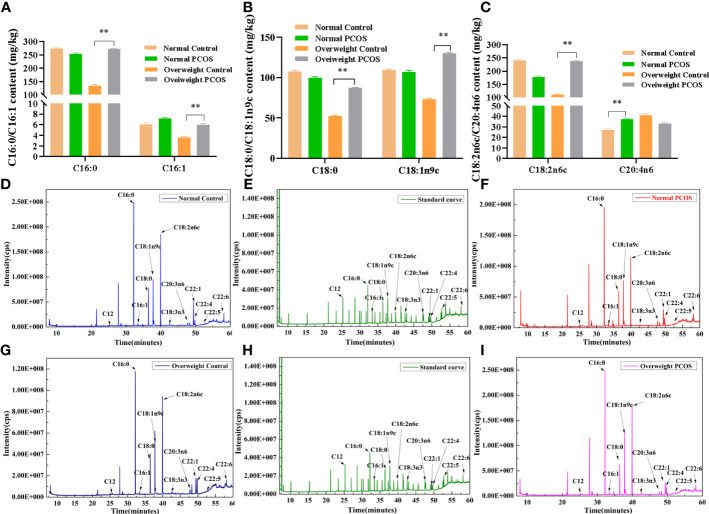
Fatty acid composition in follicular fluid. **(A–C)** C16:0、C16:1、C18:0、C18:1n9c、C18:2n6c、C20:4n6 between the overweight PCOS patients 、the overweight control subjects、the normal PCOS patients and the normal control subjects. **(D–I)** Representative GC-MS chromatograms of follicular fluid (FF) speciens from patients that underwent IVF. **(D)** FF from a normal control **(B)** FF from a normal PCOS **(E, H)** Chromatograms of 35 standard fatty acid in a standard mixture **(G)**. FF from a overweight control **(I)**. FF from an overweight PCOS. Different lowercase letters at the top of each bar denote significant differences among groups. Data represent mean ± standard error, **P < 0.01.

A total of 35 fatty acids in follicular fluid were found in different amounts in normal weight control, normal weigh PCOS, overweight control and overweight PCOS. However, some fatty acids were not detected at low levels, so a total of 18 fatty acids were detected. The content of Palmitic acid (C16:0),Palmitoleic acid (C16:1),stearic acid (C18:0), coleic acid (C18:1n9),linoleic acid(C18:2n6c) were significantly higher in the overweight PCOS women than in the overweight control women (P<0.01)(**Figures 1A–C** and **Supplementary Table 1**). In addition, the normal weight PCOS patients had a significantly higher content of arachidonic acid(C20:4n6) than the normal weight control subjects (P<0.01) (**Figure 1C** and **Supplementary Table 1**). At the same time, saturated, monounsaturated, and polyunsaturated fatty acids in follicular fluid differed among the four groups (**Supplementary Table 1**).

Complete GC-MS lipidomic datasets were obtained for the follicular fluid of normal weight control subjects, normal weight PCOS patients, overweight control subjects and overweight PCOS patients (**Figures 1D–I**).

### AA Exhibited Dose-Dependent Cytotoxicity in KGN Cells

**Figure 2:**
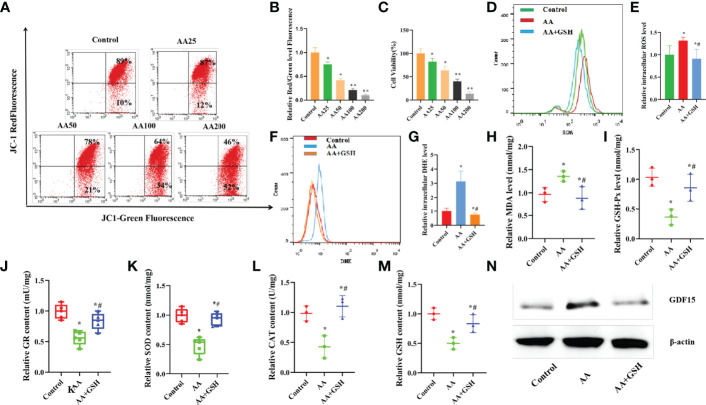
**(A–C)** AA caused KGN cells toxicity in a dose-dependent manner. **(A, B)** Mitochondrial membrane potential was decreased with increasing AA concentration. **(C)** The viability of KGN cells was decreased with increasing AA concentration. **(D–N)** AA caused damage to antioxidant capability of KGN cells. **(D, E)** AA enhanced the accumulation of the mitoSOX level. **(F, G)** AA enhanced the accumulation of the intracellular O_2-_ level. **(H–M)** AA weakened the activities of antioxidantenzymes SOD, CAT, GSH-Px and GR and the GSH content. **(N)** AA promoted the expression of GDF15 protein. Data represent mean ± standard error, ^*^P < 0.05, ^* #^P < 0.05.

As shown in **Figures 2A, B**, the mitochondrial membrane potential, as determined by measurement of the relative red and green fluorescence intensity at all time points, was decreased with increasing AA concentration after 12 h treatment. In particular, these results reveal that AA, between 0 and 50 μM, only slightly affected mitochondrial membrane potential, but between 100 and 200 μM it caused obvious impairment of mitochondrial membrane potential in KGN cells. The purpose of using the CCK-8 cell viability assay was to screen the optimal concentration of AA for the stimulation of KGN cells. AA from 0 to 25 μM did not affect the viability of KGN cells, and 50 μM only slightly affected their viability. However, AA between 100 and 200 μM showed cytotoxic effects on KGN cells. Thus, based on these two experiments, the 50 μM concentration of AA was chosen as the optimal does for subsequent experiments (**Figure 2C**).

### AA Impaired the Antioxidant Capacity of KGNs

When exposed to AA, the levels of the OS biomarkers MDA, ROS and O_2-_ levels were higher than those in the control KGN cells. After exposure to GSH, the levels of MDA, ROS and O_2-_ were restored to normal in KGN cells (**Figures 2D–H**). It is well known that OS occurs due to the limited ability of antioxidants to remove excess ROS and restore the balance between the antioxidant system and the pro-oxidant system in cells. After exposure to AA, the activities of antioxidant enzyme, including SOD, CAT,GSH-Px and GR, in KGN cells decreased, while the content of GSH also decreased(**Figures 2I–M**). Also, GSH can attenuate the oxidative damage of AA to KGN cells, due to its ability to enhance the antioxidant capacity of KGN cells and reduce the accumulation of intracellular ROS. In addition, after exposure to AA, the level of GDF15 protein increased while the level of GDF15 protein decreased after GSH supplementation (**Figure 2N**).

### AA Impaired the Secretory and Mitochondrial Functions in KGN Cells

**Figure 3:**
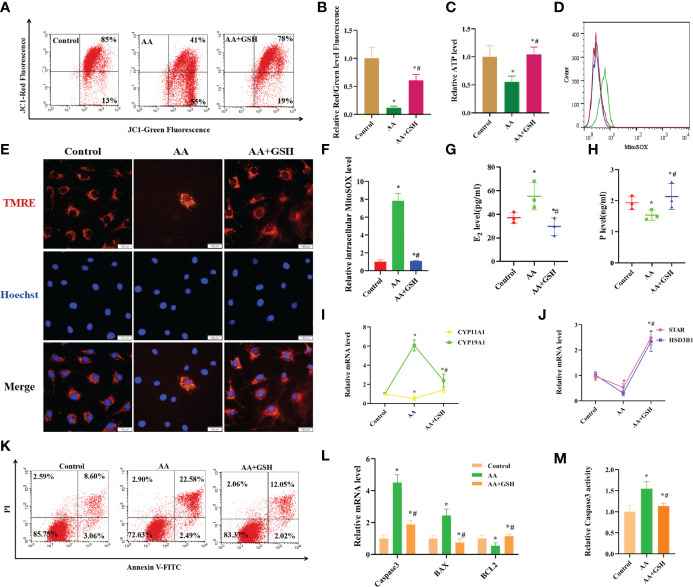
AA caused damage to secretory and mitochondrial functions of KGN cells. **(A, B)** After AA treatment, mitochondrial membrane potential was distinctly decreased. **(C)** The mitochondrial function indicators ATP level was decreased. **(D, F)** the level of intracellular mitoSOX was increased. **(E)** The TMRE level in KGN cells was measured after AA inhibition and addition of exogenous GSH. **(G–J)** KGN cells exposing to AA exhibited an aberrant secretion function including promoting estrogen secretion and inhibiting progesterone secretion concomitant with the upregulation of CYP19A1 and the downregulation of STAR, CYP11A1,HSD3B1. **(K, L)** Exposing to AA promoted KGN cells apoptosis rate together with abnormal expression of Caspase3、BAX and BCL2. **(M)** Exposing to AA promoted Caspase3 activity. Data represent mean ± standard error, ^*^P < 0.05, ^* #^P < 0.05.Bar = 100μm.

Mitochondria have previously been reported to be important organelles that control the production of energy necessary for cell survival. When exposed to AA, the ATP level and mitochondrial membrane potential decreased, while intracellular mitoSOX level increased, suggesting that mitochondrial function was impaired (**Figures 3A–F**). GSH supplementation ameliorated the AA-induced mitochondrial dysfunction. The contents of E_2_ and P in the supernatant, secreted by KGN cells *in vitro*, were determined to evaluate the effect of AA on the function of ovarian granulosa cells. KGN cells exposed to AA exhibited an impaired secretion function, including increased estrogen secretion and decreased P secretion concomitant with the upregulation of CYP19A1 and downregulation of STAR, CYP11A1,HSD3B1. Supplementation of GSH restored the secretion function of KGN cells impaired by AA (**Figures 3G–J**). TMRE is an orange-red cationic fluorescent probe that permeates cell membranes and can aggregate in intact mitochondria, but decreased depolarization or inactive mitochondrial membrane potential leads to decreased accumulation of TMRE. Staining of living cells with TMRM revealed that AA-induced mitochondrial dysfunction reduced intracellular accumulation of TMRM. Also, GSH supplementation can protect the mitochondrial function in AA-treated KGN cells (**Figure 3E**).

### AA Promotes Apoptosis in KGN Cells

It is well established that mitochondrial dysfunction is closely related to cell apoptosis. Exposure to AA enhanced the apoptosis rate of KGN cells and led to abnormal expression of Caspase3, BAX and BCL2. In contrast, supplementation of GSH decreased cell apoptosis rate and restored the normal expression levels of Caspase3, BAX and BCL2. Furthermore, exposure to AA increased Caspase3 activity, while supplementation of GSH had the opposite effect (**Figures 3K–M**).

### RNA Sequencing and Bioinformatics Analysis

**Figure 4:**
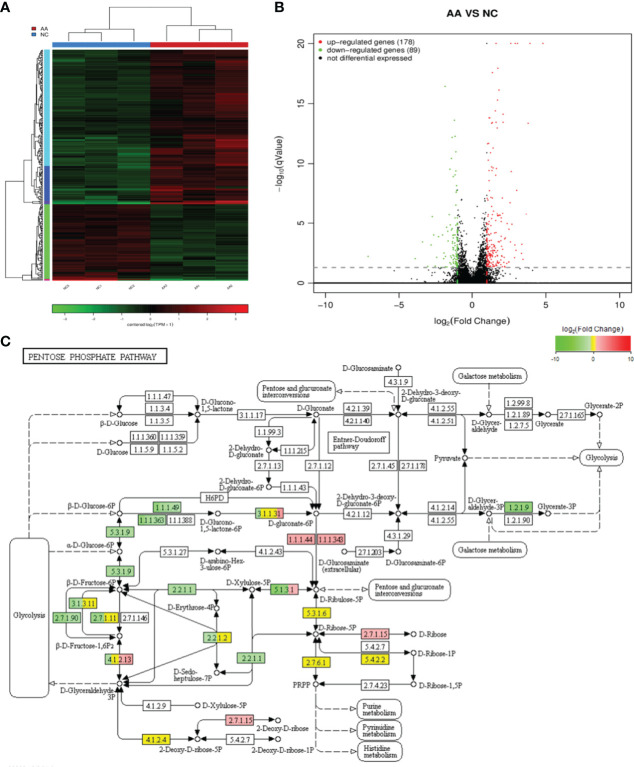
RNA sequencing and bioinformatics analysis **(A)** Hierarchical clustering heatmaps of differentially expressed genes in the KGN cells after AA treatment **(B)** Differential gene volcanic map of differentially expressed genes in the KGN cells after AA treatment. **(C)** Kyoto Encyclopedia of Genes and Genomes (KEGG) pathway classification of differentially expressed genes. KEGG pathway analysis showed that the differentially expressed genes were involved in different signaling pathways.

**Figure 5:**
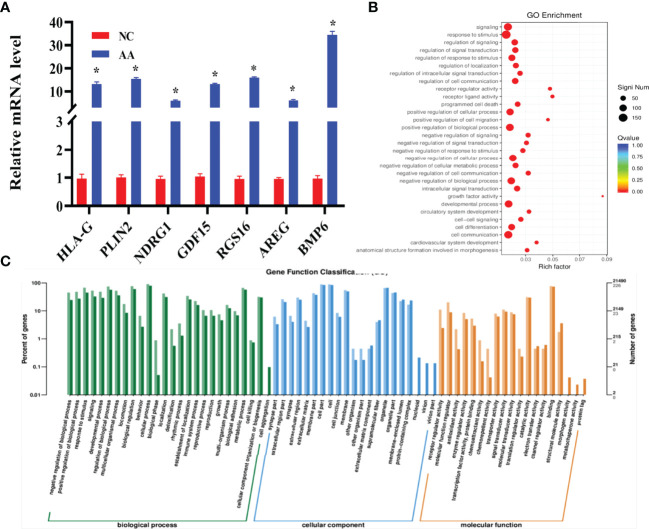
RNA sequencing and bioinformatics analysis and validation by qRT-PCR analysis **(A)** Relative expression of seven selected mRNA was quantified by qRT-PCR. Compared with the NC group, HLA-G 、PLIN、NDRG1、GDF15、RGS16、AREG and BMP6 displayed an increased expression in the AA group **(B)** GO annotation showed that the differentially expressed cells were associated with different biological processes. **(C)** Gene Ontology (GO) classification of differentially expressed cells. Data represent mean ± standard error, ^*^P < 0.05.

Analysis of gene expression profile after AA treatment was performed. A total of 267 genes were differentially expressed between the AA-treated group and normal control (NC) group (absolute log_2_ (fold change) ≥1, P<0.05). As shown in the stratified cluster heat map and volcano map in **Figures 4A, B**, among the 267 differentially expressed genes, 178 were upregulated and 89 were downregulated in the AA-treated group (**Figures 4A, B**). According to the references and our interest, 7 genes were selected from the RNA-seq data for further validation by qRT-PCR analysis (**Supplementary Table 2**).Compared with the NC group, the expression of HLA-G, PLIN1, NDRG1, GDF15, RGS16, AREG and BMP6 was increased in the AA-treated group (**Figure 5A**). The results showed that expression profiling performed by qRT-PCR was consistent with the RNA-seq analysis. Gene Ontology enrichment analysis led to the classification of the differentially expressed genes into different biological processes, such as biological adhesion, reproductive and metabolic processes (**Figures 5B, C**). The KEGG enrichment pathway analysis revealed that the various enrichment biochemical pathways associated with the differentially expressed genes included glycolysis and galactose metabolism (**Figure 4C**).

### AA Impaired the Antioxidant Capacity of KGN Cells Mediated by GDF15 as a Tolerance Response

**Figure 6:**
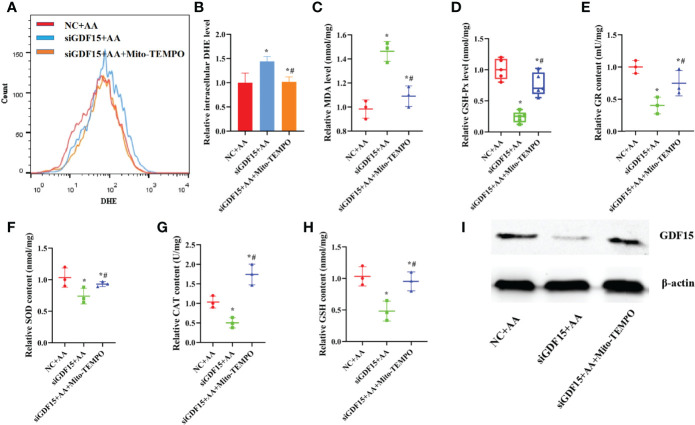
Silencing of GDF15 caused damage to antioxidant capability of KGN cells **(A, B)** silencing of GDF15 significantly promoted the levels of O_2-_ levels in KGN cells **(C–H)** Silencing of GDF15 promoted the levels of MDA and weakened the activities of antioxidant enzymes SOD, CAT, GSH-Px and GR in KGN cells and the GSH content **(I)** Silencing of GDF15 significantly decreased the level of GDF15 protein. Data represent mean ± standard error, ^*^P < 0.05, ^* #^P < 0.05.

Compared with the NC and AA-treated groups, silencing GDF15 in KGN cells significantly increased the levels of MDA and O_2-_. In addition, the silencing of GDF15 in KGN cells decreased the activities of antioxidant enzymes, including GSH-Px, GR, SOD and CAT and reduced the content of GSH (**Figures 6A–H**). Moreover, silencing *GDF15* significantly reduced GDF15 protein levels (**Figure 6I**). However, the addition of Mito-TEMPO significantly reduced the increased levels of MDA and O_2-_ in KGN cells. Additionally, it restored the activities of GSH-Px, GR, SOD and CAT and increased the content of GSH. Also, the protein level of GDF15 increased after treatment with Mito-TEMPO.

**Figure 7:**
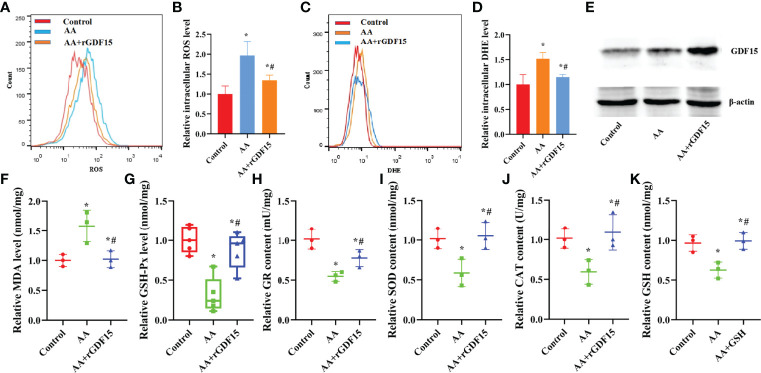
The supplementation of exogenous rGDF15 restored antioxidant capability of KGN cells caused by AA. **(A–D)** Pretreatment of rGDF15 reduced the levels of ROS and O_2-_ levels in KGN cells. **(E)**The supplementation of rGDF15 increased the level of GDF15 protein after exposed to AA. **(F–K)** Exogenous supplementation of rGDF15 reduced the levels of MDA and restored the activities of antioxidant enzymes SOD, CAT, GSH-Px and GR in KGN cells and advanced the GSH content caused by AA. Data represent mean ± standard error, ^*^P < 0.05, ^* #^P < 0.05.

Besides, pretreatment with rGDF15 not only reduced the levels of MDA, ROS and O_2-_ in KGN cells (**Figures 7A–D**), but also restored the activities of antioxidant enzymes such as GSH-Px, GR, SOD and CAT and increased the content of GSH induced by AA (**Figures 7F–K**). Furthermore, the addition of rGDF15 increased GDF15 protein levels after exposure to AA (**Figure 7E**).

### AA Caused Damage to the Secretory and Mitochondrial Function of KGNs, Which Was Inhibited by Upregulating GDF15 Expression as a Tolerance Response

**Figure 8:**
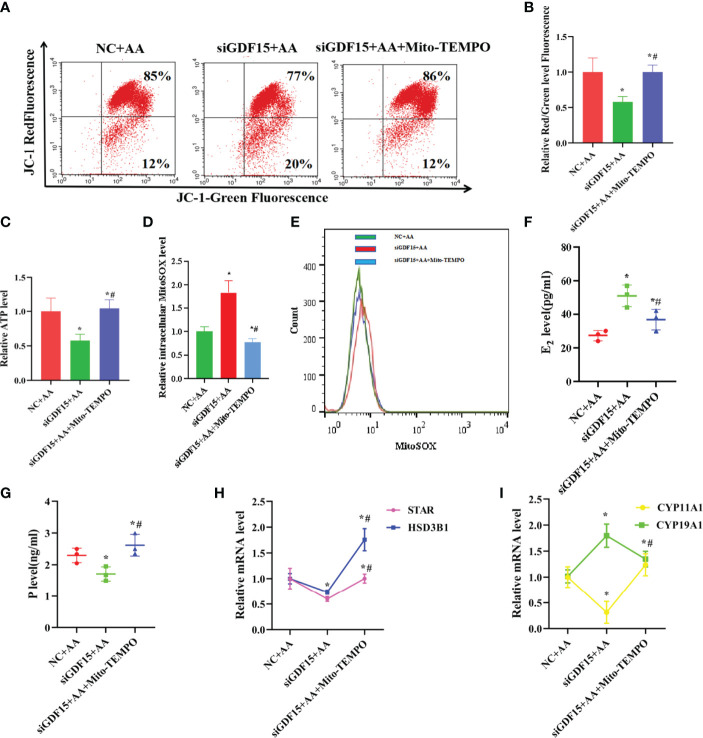
Silencing of GDF15 caused damage to the secretory and mitochondrial function and increased apoptosis rate of KGN cells **(A, B)** silencing of GDF15 promoted mitochondrial dysfunction and intracellular weakened ATP level. **(C)** when silencing of GDF15, the mitochondrial function indicators ATP level was decreased. **(D, E)** when silencing of GDF15,the level of intracellular mitoSOX was increased. **(F–I)** silencing of GDF15 exhibited an aberrant secretion function including promoting estrogen secretion and inhibiting progesterone secretion concomitant with the upregulation of CYP19A1 and the downregulation of STAR, CYP11A1,HSD3B1. Data represent mean ± standard error, ^*^P < 0.05, ^* #^P < 0.05.

GDF15 encodes a secretory ligand of the TGF-β (transforming growth factor -β) superfamily of proteins. Increased protein levels are associated with disease states, such as tissue hypoxia, inflammation, acute injury and OS. While silencing of GDF15 promoted mitochondrial dysfunction and reduced intracellular ATP levels, treatment with Mito-TEMPO resulted in the opposite effects, indicating that the expression level of GDF15 had a protective function in mitochondria as a tolerance response (**Figures 8A–E**). Silencing of GDF15 in KGN cells also led to abnormal secretion E_2_ and P and abnormal expression levels of STAR, HSD3B1,CYP11A1 and CYP19A1 (**Figures 8F–I**). Treatment with Mito-TEMPO counteracted the above effects induced by AA.

**Figure 9:**
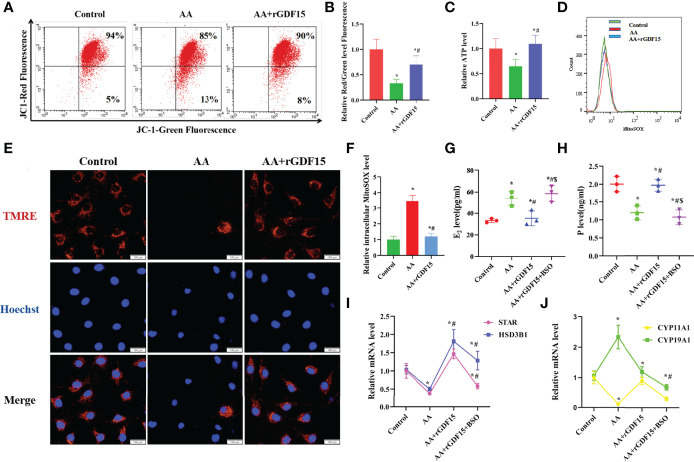
Supplementation with exogenous rGDF15 restored secretory and mitochondrial functions of KGN cells caused by AA. **(A, B)** Supplementation with exogenous rGDF15 restored mitochondrial membrane potential **(C)** Supplementation with exogenous rGDF15 improved the ATP level of KGN cells. **(D, F)** Replenishment of rGDF15 decreased level of intracellular mitoSOX. **(E)** The TMRE level in KGN cells was measured after AA inhibition and addition of exogenous rGDF15 **(G–J)** Replenishment of rGDF15 ameliorated the secretory function produced by AA, restoring the secretory of E_2_ and P along with the aberrant expression of STAR, CYP11A1,HSD3B1 and CYP19A1. When supplementation with exogenous GSH synthesis inhibitor BSO hinder this amelioration of secretory function, the rescue of the rGDF15 on E_2_ and P secretion was interrupted together with the attenuated ability of in restoring STAR, CYP11A1,HSD3B1and CYP19A1 mRNA levels. Data represent mean ± standard error, ^*^P < 0.05, ^* #^P < 0.05, ^* #$^P < 0.05,Bar = 100μm.

Supplementation with exogenous rGDF15 restored mitochondrial function, as indicated by the increase in the mitochondrial membrane potential and ATP levels and the decrease in mitoSOX (**Figures 9A–F**). In addition, the supplementation of rGDF15 improved the secretion function impaired by AA, restored the secretion of E_2_ and P and the expression of the mRNA levels of STAR, CYP11A1,HSD3B1 and CYP19A1 to normal levels (**Figures 9F–I**). When GSH synthesis was inhibited by treatment with the exogenous GSH synthesis inhibitor buthionine sulfoximine (BSO) the rGDF15 effects on the improvement of the secretory function, rescue of the secretion of E_2_ and P and the expression of the mRNA levels of STAR, CYP11A1, HSD3B1 and CYP19A1 (**Figures 9G–J**). Moreover, staining of living cells with TMRE revealed that AA-induced mitochondrial dysfunction reduced intracellular accumulation of TMRM. Overall, these experiments revealed that supplementation of exogenous rGDF15 can protect the mitochondrial function in AA-treated KGN cells (**Figure 9E**).

### AA Promoted Apoptosis in KGN Cells, Which Was Counteracted by Upregulating GDF15 Expression as a Tolerance Response

**Figure 10:**
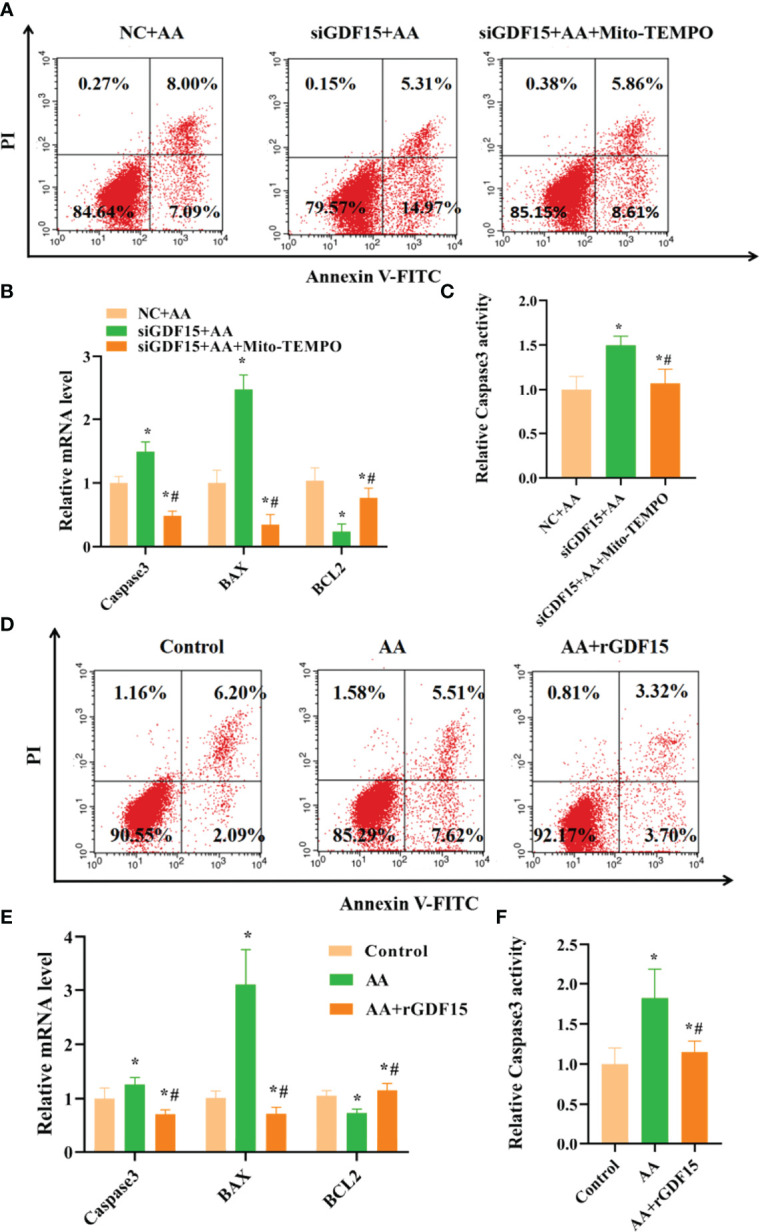
Silencing of GDF15 increased apoptosis rate of KGN cells and supplementation with exogenous rGDF15 decreased apoptosis rate of KGNs caused by AA. **(A, B)** Silencing of GDF15 increased KGN cells apoptosis rate together with abnormal expression of Caspase3、BAX and BCL2. **(C)** Silencing of GDF15 promoted Caspase3 activity. **(D, E)** Supplementation with exogenous rGDF15 reduced KGN cells apoptosis rate together with abnormal expression of Caspase3、BAX and BCL2. **(F)** Supplementation with exogenous rGDF15 restored Caspase3 activity. Data represent mean ± standard error, ^*^P < 0.05, ^* #^P < 0.05.

In this study, silencing of GDF15 increased the apoptosis rate in KGN cells and Caspase3 activity, and also led to the aberrant expression of Caspase3、BAX and BCL2 (**Figures 10A–C**). In contrast, treatment with Mito-TEMPO decreased apoptosis rate in KGN cells and Caspase3 activity, and restored the normal expression of levels of Caspase3, BAX and BCL2.

Supplementation with exogenous rGDF15 had a protective effect on AA-induced apoptosis in KGN cells and reduced Caspase3 activity and restored the normal expression levels of Caspase3, BAX and BCL2 after exposure to AA (**Figures 10D–F**).

## Discussion

PCOS is a disease associated with metabolic and endocrine abnormalities (17). In this study, the serum testosterone level and duration of infertility of PCOS patients on days 3-5 of the menstrual cycle was significantly higher than that of the control group (P<0.01). In addition, the E_2_ and testosterone level of PCOS patients was significantly higher than that of the control group on the day of administration (P<0.05). Another earlier study also reported higher levels of testosterone in follicular fluid of patients with PCOS than that of healthy subjects (16).In patients with ovulation stimulation, pituitary suppression and exogenous follicle-stimulating hormone (FSH) treatment did not alter this difference (16). Given these findings, it is necessary to study the consequences of intra-follicular hyperandrogenemia. Although there was no significant difference in laboratory outcomes between the two groups, the embryo implantation rate was lower in the PCOS group than in the normal control group. This is attributed to the poorly of quality oocytes and decreased the embryo implantation rate in PCOS patients, even though they produced more oocytes after ovulation induction, which was consistent with other research results (18).

It has been suggested that follicular fluid provides a microenvironment for oocyte development and maturation and its components may influence oocyte development (7, 9, 19, 20). We tested a total of 35 fatty acids, but only 18 were detected due to low levels of some fatty acids in normal weight control, normal weigh PCOS, overweight control and overweight PCOS. The content of Palmitic acid (C16:0), Palmitoleic acid (C16:1), stearic acid (C18:0), coleic acid (C18:1n9), linoleic acid(C18:2n6c) were significantly higher in the overweight PCOS women than in the overweight control women.Fatty acids are mainly divided into saturated fatty acids(SFA), monounsaturated fatty acids (MUFA) and polyunsaturated fatty acids (PUFA).

Different fatty acid compositions have different effects on body metabolism. Compared with monounsaturated fatty acids and polyunsaturated fatty acids, saturated fatty acid-loaded meals can increase the level of oxidative stress in the body. It is well known that excessive intake of saturated fatty acids such as Palmitic acid (PA)and stearic acid can increase the level of cholesterol in the body, have an important impact on cardiovascular and cerebrovascular diseases (21).Oxidative stress caused by PA overloading is initial development of endothelial dysfunction. At the same time, excessive saturated fatty acids promote the production of inflammatory factors in adipose tissue, which has a pro-inflammatory effect, while inflammation factors aggravate cellular oxidative stress damage (22).

Palmitoleic acid, and oleic acid are common monounsaturated fatty acids in the human body. They have been reported to induce cytotoxic responses called “Lipotoxicity” (23). The reported lipotoxicity caused by palmitoleic acid and oleic acid is mediated by increased levels of ROS or oxidative stress, and several mechanisms are involved, including enhancements O_2-_, which is associated with complex I, is generated by β-oxidation, interferes with various enzymatic processes, and is associated with components of the respiratory chain, etc (24).

PUFA is a subtype of fatty acid. According to the position of the first carbon double bond from the methyl carbon, it can be divided into n-3, n-6, n-7, and n-9 series (25). At present, the most studied is n-3. and n-6 series. The parent of n-6 series PUFAs is linoleic acid (LA), and n-6 fatty acids have pro-inflammatory and oxidative stress-promoting effects (26). Both n-3PUFAs and n-6PUFAs can improve inflammation-related risk factors in PCOS, but higher intake of n-6PUFAs may weaken the effect of n-3PUFAs. Therefore, polyunsaturated fatty acids may have effects on the body through inflammatory and oxidative stress signaling (27).

In addition, the normal weight PCOS patients had a significantly higher content of arachidonic acid (C20:4n6) than the normal weight control subjects. AA is the most abundant and widely distributed unsaturated fatty acid in the human body. AA derived metabolites, particularly prostaglandin (PG), play a key role in oocyte maturation, cumulus expansion, and ovulation. In addition, AA can produce a class of eicosanoids with high biological activity, which are mainly used as an indicator of OS (28). In this study, it was found that after hyperstimulation AA level in the follicular fluid of normal weight PCOS group was higher than that in the normal control group. In order to determine whether high AA in follicular fluid has an effect on the function of granulosa cells, KGN cells were used for *in vitro* validation studies.

This study revealed that AA was cytotoxic to KGN cells in a dose-dependent manner. Therefore, we chose AA at a concentration of 50 μM as the experimental dose in this study. Additionally, we also found that AA impaired the secretory and mitochondrial functions in KGN cells. After exposure to AA, OS biomarkers, including MDA, ROS and O_2-_ levels were found increased in KGN cells. Also, after exposure to AA, the activities of the antioxidant enzymes GSH-Px, GR, SOD and CAT, as well as the GSH content, were decreased in KGN cells. Several studies have shown that excessive production of ROS leads to increased lipid peroxidation and production of MDA, a marker of OS (29). MDA is a harmful irritant that causes protein misfolding and increases the severity of OS responses (30, 31). Excessive ROS production in OS-stimulate cells also results in reduced total antioxidant capacity and antioxidant activity of antioxidant enzymes, such as SOD, CAT,GSH-Px and GR (32). In addition, AA also reduces GSH content, which is an important antioxidant that scavenge free radicals in the body.

Moreover, the production of excessive ROS during OS can disrupt the mitochondrial membrane potential balance, resulting in mitochondrial damage (33–35). When cells are exposed to AA, mitochondrial function indicators, such as ATP level and mitochondrial membrane potential are distinctly decreased, while the level of intracellular mitoSOX is increased. In turn, the production of ROS is also induced by inhibiting the activity of complex I and III enzymes in the mitochondrial respiratory chain and increasing the fluidity of mitochondrial membranes. In fact, studies have found that ROS production in rat cardiomyocyte mitochondria was positively linearly correlated with the degree of inhibition of complex III enzymes (36).

Estrogen and P, which are two major hormones produced in ovarian granulosa cells, are involved in important reproductive metabolic activities in the human body. KGN cells exposed to AA exhibited an aberrant secretion function including enhanced estrogen secretion and reduced P secretion concomitant with the upregulation of CYP19A1 and the downregulation of STAR, CYP11A1 and HSD3B1, which play important part in the synthesis of steroid hormones. One of the most important functions of ovarian granulosa cells is the synthesis and secretion of steroid hormones necessary for female reproduction. The synthesis of these hormones is regulated by gonadotropins and various nutritional factors in the follicular fluid microenvironment (37).Most steroid synthetases are members of the cytochrome superfamily. In membrane cells, STAR is responsible for the transport of cholesterol into the mitochondria and is involved in the first step of steroid synthesis (38). In addition, cholesterol is converted to P by CYP11A1 (cholesterol side chain lyase) and HSD3B1 (3βhydroxy steroid dehydrogenase) (39).In turn, P is converted to androgen by carbon chain lyase and to estrogen by 17α2 hydroxylase (P450 17α) and C17,20 carbon chain lyase. Also, androgens enter granulosa cells and are converted to estrogen by CYP19A1 (40). Some studies have shown that changes in the expression of steroidogenic enzymes affect steroid hormone production (41).Therefore, exposure to AA led to an aberrant secretion function including reduced P secretion concomitant with the upregulation of CYP19A1 and increased estrogen secretion and the downregulation of STAR, CYP11A1 and HSD3B1.OS can lead to mitochondrial damage and severe mitochondrial damage can lead to apoptosis in KGN cells (42). Flow cytometry analysis results showed that the apoptosis rate was significantly increased after AA treatment and supplementation of GSH resulted in reduced apoptosis rate. Furthermore, BCL2 and BAX are important anti-apoptotic and pro-apoptotic proteins, respectively, located on the surface of the mitochondrial membrane. The ratio of BCL2 and BAX can reflect the apoptotic state of cells and is considered to be an important indicator of mitochondrial permeability damage. In addition, the apoptosis executor Caspase3 is an important pro-apoptotic protein (43).

This study also performed transcriptome sequencing on AA-treated KGN cells to further investigate the downstream genes affected by AA and its mechanism of action. Transcriptome sequencing identified a total of 267 differentially expressed genes (absolute log_2_(fold change)≥1, P <0.05), of which 178 genes were upregulated and 89 genes were downregulated in the AA-treated group. The function of downstream target genes of AA was further analyzed by GO enrichment analysis and KEGG pathway enrichment analysis using bioinformatics software. The KEGG pathway enrichment analysis revealed that the differential expressed genes were mainly related to glycolysis metabolism and galactose metabolism and other metabolic pathways. On the other hand, according to GO enrichment analysis results of differentially expressed genes, the differentially expressed genes are mainly related to biological adhesion, reproductive process, and metabolic process. Numerous previous studies have shown that among these pathways, metabolic process, especially lipid metabolism, is a biological process closely related to PCOS. Nearly half of PCOS patients are associated with obesity, and circulating total free fatty acids are often elevated.

GDF15 has a protective effect against tissue damage and inflammation, acute tissue damage can lead to compensatory increase in GDF15 levels (46). Additionally, GDF15 has a protective effect against genetic and diet-related obesity (47).GDF15 overexpression improves insulin sensitivity, and its knockdown enhances blood glucose levels in diabetic rat models, supporting its beneficial effect on metabolic diseases (48). Studies have shown that AA effectively induces OS and then exerts pro-apoptotic effects (49). Our experimental results showed that AA induced OS in KGN cells, but rGDF15 effectively prevented the damage effect of AA on KGN cells. In addition, silencing of GDF15 exacerbated the AA-induced cell damage. These findings show that GDF15 plays an important role in AA-induced OS in KGN cells. Therefore, we hypothesize that AA induced ROS and upregulated GDF15 to deal with the OS, thereby, protecting against the ROS-mediated impairment of the mitochondrial membrane potential, which effectively alleviated mitochondrial damage and then exerted anti-apoptotic effects (50, 51).

**Figure 11:**
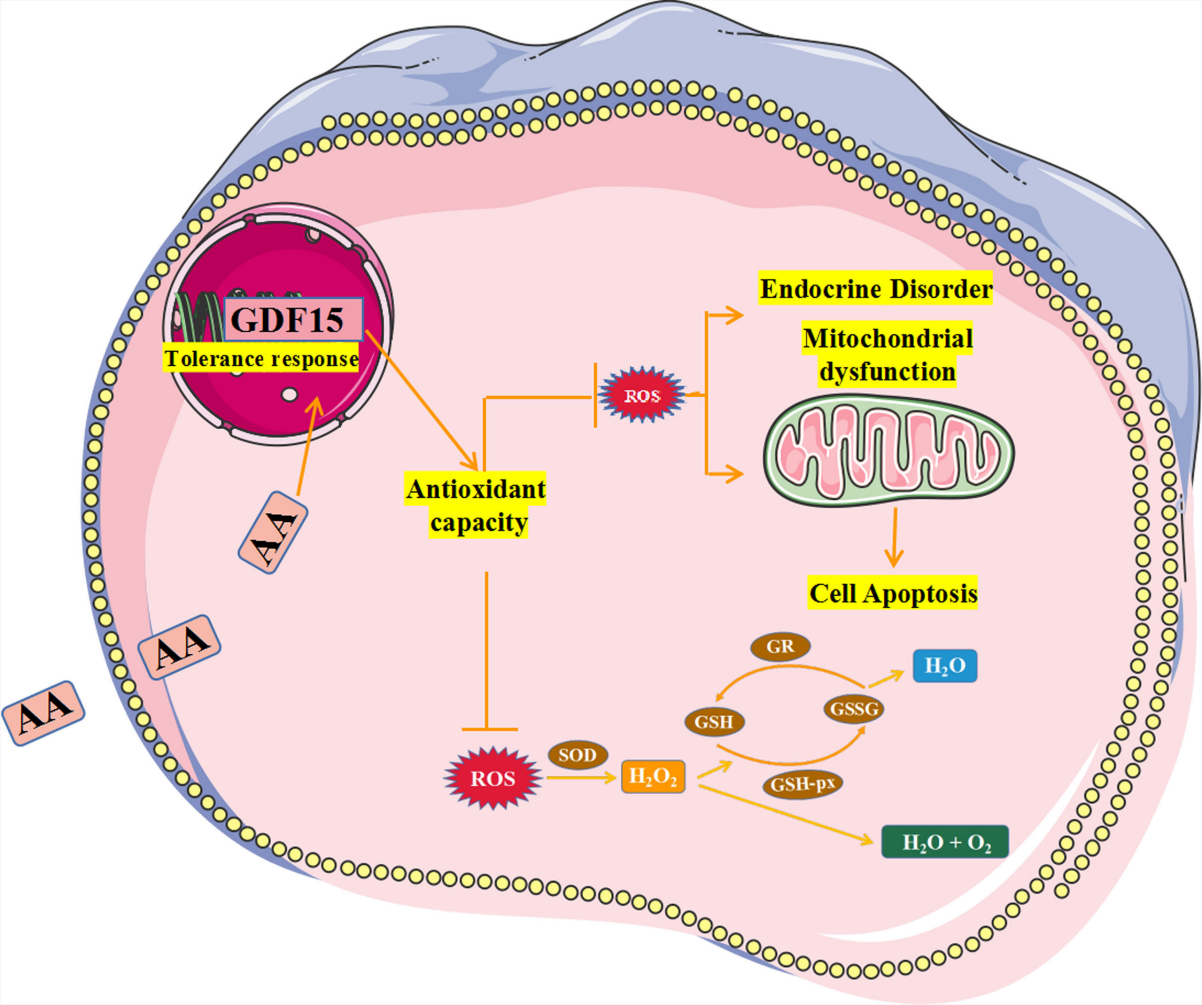
Schematic summarizing the effect and mechanism of AA inducing KGN cells oxidative stress. AA weakened the KGN cells antioxidant capability by increasing ROS level and inhibiting activity of antioxidant enzymes.In addition, AA caused damage to secretory and mitochondrial functions of KGN cells and promoted cell apoptosis. Therefore, AA induces OS in KGN cells and upregulates the expression of GDF15 as a protective response against OS.

In conclusion, this study found that AA induces OS in KGN cells and upregulates the expression of GDF15 as a protective response against OS (**Figure 11**).

## Data Availability Statement

The datasets presented in this study can be found in online repositories. The names of the repository/repositories and accession number(s)can be found below: SRA, PRJNA812712.

## Ethics Statement

The studies involving human participants were reviewed and approved by The Ethics Committee of the Second Hospital of Jilin University (Jilin, China). The patients/participants provided their written informed consent to participate in this study. Written informed consent was obtained from the individual(s) for the publication of any potentially identifiable images or data included in this article.

## Author Contributions

All of the authors contributed to the conception of the article. YM and LZ performed material data collection. YG and YW performed analysis preparation. Conception and design of the study were performed by YM and YX. The first draft of the manuscript was prepared by YM. LZ, YW, and YG performed subsequent amendments. YX revised the manuscript. All authors read and approved the submitted version final manuscript.

## Conflict of Interest

The authors declare that the research was conducted in the absence of any commercial or financial relationships that could be construed as a potential conflict of interest.

## Publisher’s Note

All claims expressed in this article are solely those of the authors and do not necessarily represent those of their affiliated organizations, or those of the publisher, the editors and the reviewers. Any product that may be evaluated in this article, or claim that may be made by its manufacturer, is not guaranteed or endorsed by the publisher.

## Supplementary Material

Supplementary Table 1 Comparison of fatty acid levels between the overweight PCOS patients 、the overweight control subjects、the normal PCOS subjects and the normal control subjects.

Supplementary Table 2 Analysis of differences in gene expression between the two groups.

## References

[1] Homburg R Berkowitz D Levy T Feldberg D Ashkenazi J Ben-Rafael Z. *In Vitro* Fertilization and Embryo Transfer for the Treatment of Infertility Associated With Polycystic Ovary Syndrome. Fertil Steril (1993) 60(5):858–63. doi: 10.1016/s0015-0282(16)56287-6 8224271

[2] Heijnen EMEW Eijkemans MJC Hughes EG Laven JSE Macklon NS Fauser BCJM. A Meta-Analysis of Outcomes of Conventional IVF in Women With Polycystic Ovary Syndrome. Hum Reprod Update (2006) 12(1):13–21. doi: 10.1093/humupd/dmi036 16123051

[3] Kim YS Kim MS Lee SH Choi BC Lim JM KY C. Proteomic Analysis of Recurrent Spontaneous Abortion: Identification of an Inadequately Expressed Set of Proteins in Human Follicular Fluid. Proteomics (2006) 6(11):3445–54. doi: 10.1002/pmic.200500775 16637005

[4] Von Wald T Monisova Y Hacker MR Yoo SW Penzias AS Reindollar RR. Age-Related Variations in Follicular Apolipoproteins may Influence Human Oocyte Maturation and Fertility Potential. Fertil Steril (2010) 93(7):2354–61. doi: 10.1016/j.fertnstert.2008.12.129 19230882

[5] Huang R Xue X Li S Wang Y Sun Y Liu W. Alterations of Polyunsaturated Fatty Acid Metabolism in Ovarian Tissues of Polycystic Ovary Syndrome Rats. J Cell Mol Med (2018) 22(7):3388–96. doi: 10.1111/jcmm.13614 PMC6010729 29602230

[6] Revelli A Piane LD Casano S Molinari E Massobrio M Rinaudo P. Follicular Fluid Content and Oocyte Quality: From Single Biochemical Markers to Metabolomics. Reprod Biol Endocrinol (2009) 7(1). doi: 10.1186/1477-7827-7-40 PMC2685803 19413899

[7] Jungheim ES Macones GA Odem RR Patterson BW Lanzendorf SE Ratts VS. Associations Between Free Fatty Acids, Cumulus Oocyte Complex Morphology and Ovarian Function During *In Vitro* Fertilization. Fertil Steril (2011) 95(6):1970–4. doi: 10.1016/j.fertnstert.2011.01.154 PMC3080431 21353671

[8] Nehra D Le HD Fallon EM Carlson SJ Woods D White YA. Prolonging the Female Reproductive Lifespan and Improving Egg Quality With Dietary Omega-3 Fatty Acids. Aging Cell (2012) 11(6):1046–54. doi: 10.1111/acel.12006 PMC5624332 22978268

[9] Kazemi A Ramezanzadeh F Nasr-Esfahani MH Saboor Yaraghi AA Ahmadi. Does Dietary Fat Intake Influence Oocyte Competence and Embryo Quality by Inducing Oxidative Stress in Follicular Fluid? Iran J Reprod Med (2013) 11(12):1005–12. doi: 10.1097/AOG.0000000000000023 PMC3941410 24639727

[10] Tokugawa Y Kunishige I Kubota Y Shimoya K Nobunaga T Kimura T. Lipocalin-Type Prostaglandin D Synthase in Human Male Reproductive Organs and Seminal Plasma. Biol Reprod (1998) 58(2):600–7. doi: 10.1095/biolreprod58.2.600 9475419

[11] Marions L Danielsson KG. Expression of Cyclo-Oxygenase in Human Endometrium During the Implantation Period. Mol Hum Reprod (1999) 5(10):961–5. doi: 10.1093/molehr/5.10.961 10508225

[12] Lim H Gupta RA Ma WG Paria BC Moller DE Morrow JD. Cyclo-Oxygenase-2-Derived Prostacyclin Mediates Embryo Implantation in the Mouse *via* PPARdelta. Genes Dev (1999) 13(12):1561–74. doi: 10.1101/gad.13.12.1561 PMC316805 10385625

[13] Rotterdam EA-SPCWG. Revised 2003 Consensus on Diagnostic Criteria and Long-Term Health Risks Related to Polycystic Ovary Syndrome. Fertil Steril (2004) 81(1):19–25. doi: 10.1016/j.fertnstert.2003.10.004 14711538

[14] Endocrinology Subgroup and Expert Panel Chinese Society of Obstetrics and Gyneocology Chinese Medical Association. Chinese Guideline for Diagnosis and Management of Polycystic Ovary Syndrome. Zhonghua Fu Chan Ke Za Zhi (2018) 53(1):2–6. doi: 10.3760/cma.j.issn.0529-567X.2018.01.002 29374878

[15] Rosenfield RL Ehrmann DA. The Pathogenesis of Polycystic Ovary Syndrome (PCOS): The Hypothesis of PCOS as Functional Ovarian Hyperandrogenism Revisited. Endoc Rev (2016) 37(5):467–520. doi: 10.1210/er.2015-1104 PMC5045492 27459230

[16] Yang F Ruan YC Yang YJ Wang K Liang SS Han YB. Follicular Hyperandrogenism Downregulates Aromatase in Luteinized Granulosa Cells in Polycystic Ovary Syndrome Women. Reproduction (2015) 150:289–96. doi: 10.1530/REP-15-0044 26199450

[17] Lim SS Kakoly NS Tan JWJ Fitzgerald G Bahri Khomami M Joham AE. Metabolic Syndrome in Polycystic Ovary Syndrome: A Systematic Review, Meta-Analysis and Meta-Regression. Obes Rev (2019) 20(2):339–52. doi: 10.1111/obr.12762 30339316

[18] Robker RL Akison LK Bennett BD Thrupp PN Chura LR Russell DL. Obese Women Exhibit Differences in Ovarian Metabolites, Hormones, and Gene Expression Compared With Moderate-Weight Women. J Clin Endocrinol Metab (2009) 94(5):1533–40. doi: 10.1210/jc.2008-2648 19223519

[19] Leroy JLMR Rizos D Sturmey R Bossaert P Gutierrez-Adan A Van Hoeck V. Intrafollicular Conditions as a Major Link Between Maternal Metabolism and Oocyte Quality: A Focus on Dairy Cow Fertility. Reproduction. Fertil Dev (2012) 24(1):1. doi: 10.1071/rd11901 22394712

[20] Li R Zhang Q Yang D Li S Lu S Wu X. Prevalence of Polycystic Ovary Syndrome in Women in China: A Large Community-Based Study. Hum Reprod (2013) 28(9):2562–9. doi: 10.1093/humrep/det262 23814096

[21] Hu D Xu Y Xie J Sun C Zheng X Chen W. Systematic Evaluation of Phenolic Compounds and Protective Capacity of a New Mulberry Cultivar J33 Against Palmitic Acid-Induced Lipotoxicity Using a Simulated Digestion Method. Food Chem (2018) 258:43–50. doi: 10.1016/j.foodchem.2018.03.049 29655752

[22] Schönfeld P Wojtczak L. Fatty Acids as Modulators of the Cellular Production of Reactive Oxygen Species. Free Radical Biol (2008) 45:231–41. doi: 10.1016/j.freeradbiomed.2008.04.029 18482593

[23] Harman D. Aging: A Theory Based on Free Radical and Radiation Chemistry. J Gerontol (1956) 11:298–300. doi: 10.1093/geronj/11.3.298 13332224

[24] Sugamura K Keaney JF Jr. Reactive Oxygen Species in Cardiovascular Disease. Free Radical Biol Med (2011) 51:978–92. doi: 10.1016/j.freeradbiomed.2011.05.004 PMC3156326 21627987

[25] Liu YH Li XY Chen CY Zhang HM Kang JX. Omega-3 Fatty Acid Intervention Suppresses Lipopolysaccharide-Induced Inflammation and Weight Loss in Mice. Mar Drugs (2015) 13(2):1026–36. doi: 10.3390/md13021026 PMC4344616 25689565

[26] Hayashi S Satoh Y Ogasawara Y Dairi T. Recent Advances in Functional Analysis of Polyunsaturated Fatty Acid Synthases. Curr Opin Chem Biol (2020) 59:30–6. doi: 10.1016/j.cbpa.2020.04.015 32442859

[27] Zhang HT Shan L Bi YP. Functional Relationship Between N-6 and N-3 Polyunsaturated Fatty Acids in Human and Animal. Shandong Agric Sci (2006) 2:115–20. doi: 10.3969/j.issn.1001-4942.2006.02.044

[28] SchNfeld P Wojtczak L. Fatty Acids Decrease Mitochondrial Generation of Reactive Oxygen Species at the Reverse Electron Transport But Increase it at the Forward Transport. Biochim Biophys Acta (BBA) - Bioenerget (2007) 1767(8):1032–40. doi: 10.1016/j.bbabio.2007.04.005 17588527

[29] Frijhoff J Winyard PG Zarkovic N Davies SS Stocker R Cheng D. Clinical Relevance of Biomarkers of Oxidative Stress. Antiox Redox Signaling (2015) 23(14):1144–70. doi: 10.1089/ars.2015.6317 PMC4657513 26415143

[30] Wang L Zhang M Fang Z Bhandari B. Gelation Properties of Myofibrillar Protein Under Malondialdehyde-Induced Oxidative Stress. J Sci Food Agric (2017) 97:50–7. doi: 10.1002/jsfa.7680 26916602

[31] Lv H Liu Q Wen Z Feng H Deng X Ci X. Xanthohumol Ameliorates Lipopolysaccharide (LPS)-Induced Acute Lung Injury *via* Induction of AMPK/Gsk3β-Nrf2 Signal Axis. Redox Biol (2017) 12:311–24. doi: 10.1016/j.redox.2017.03.001 PMC5345976 28285192

[32] Romero A Novoa B Figueras A. The Complexity of Apoptotic Cell Death in Mollusks: An Update. Fish Shellfish Immunol (2015) 46(1):79–87. doi: 10.1016/j.fsi.2015.03.038 25862972

[33] Yang X Feng L Zhang Y Hu H Shi Y Liang S. Cytotoxicity Induced by Fine Particulate Matter (PM2.5) *via* Mitochondria-Mediated Apoptosis Pathway in Human Cardiomyocytes. Ecotoxicol Environ Saf (2018) 161:198–207. doi: 10.1016/j.ecoenv.2018.05.092 29885615

[34] Miller WL. Steroidogenesis: Unanswered Questions. Trends Endocrinol Metab (2017) 28(11):771–93. doi: 10.1016/j.tem.2017.09.002 29031608

[35] Hattori K Orisaka M Fukuda S Tajima K Yamazaki Y Mizutani T. Luteinizing Hormone Facilitates Antral Follicular Maturation and Survival *via* Thecal Paracrine Signaling in Cattle. Endocrinology (2018) 159(6):2337–47. doi: 10.1210/en.2018-00123 29668890

[36] Eimerl S Orly J. Regulation of Steroidogenic Genes by Insulin-Like Growth Factor-1 and Follicle-Stimulating Hormone: Differential Responses of Cytochrome P450 Side-Chain Cleavage, Steroidogenic Acute Regulatory Protein, and 3β-Hydroxysteroid Dehydrogenase/Isomerase in Rat Granulosa Cells1. Biol Reprod (2002) 67(3):900–10. doi: 10.1095/biolreprod.101.002170 12193401

[37] Rodgers RJ Rodgers HF Hall PF Waterman MR Simpson ER. Immunolocalization of Cholesterol Side-Chain-Cleavage Cytochrome P-450 and 17 Alpha-Hydroxylase Cytochrome P-450 in Bovine Ovarian Follicles. J Reprod Fertil (1986) 78(2):627. doi: 10.1530/jrf.0.0780627 3543333

[38] Bley MA Saragüeta PE Bara?Ao JL. Concerted Stimulation of Rat Granulosa Cell Deoxyribonucleic Acid Synthesis by Sex Steroids and Follicle-Stimulating Hormone. J Steroid Biochem Mol Biol (1997) 62(1):0–19. doi: 10.1016/s0960-0760(97)00021-6 9366494

[39] Lavoie HA King SR. Transcriptional Regulation of Steroidogenic Genes: STARD1, CYP11A1 and HSD3B. Exp Biol Med (2009) 234(8):880–907. doi: 10.3181/0903-MR-97 19491374

[40] Legro RS Kunselman AR Dunaif A. Prevalence and Predictors of Dyslipidemia in Women With Polycystic Ovary Syndrome. Am J Med (2001) 111(8):607–13. doi: 10.1016/S0002-9343(01)00948-2 11755503

[41] Payne AH Hales DB. Overview of Steroidogenic Enzymes in the Pathway From Cholesterol to Active Steroid Hormones. Endoc Rev (2003) 6:947. doi: 10.1210/er.2003-0030 15583024

[42] Chekir C Nakatsuka M Kamada Y Noguchi S Sasaki A Hiramatsu Y. Impaired Uterine Perfusion Associated With Metabolic Disorders in Women With Polycystic Ovary Syndrome. Acta Obstetricia Gynecol Scandinavica (2005) 84:189–95. doi: 10.1111/j.0001-6349.2005.00678.x 15683382

[43] Kan X Liu J Chen Y Guo W Xu D Cheng J. Myricetin Protects Against H2O2 -Induced Oxidative Damage and Apoptosis in Bovine Mammary Epithelial Cells. J Cell Physiol (2020) 236(4):2684–95. doi: 10.1002/jcp.30035 32885418

[44] Sun Y Lian M Lin Y Xu B Li Y Wen J. Role of P-Mkk7 in Myricetin-Induced Protection Against Intestinal Ischemia/Reperfusion Injury. Pharmacol Res (2017) 129:432–42. doi: 10.1016/j.phrs.2017.11.011 29154988

[45] Nakayasu ES Syed F Tersey SA Gritsenko MA Mitchell HD Chan CY. Comprehensive Proteomics Analysis of Stressed Human Islets Identifies GDF15 as a Target for Type 1 Diabetes Intervention. Cell Metab (2020) 31(2):363–374.e366. doi: 10.1016/j.cmet.2019.12.005 31928885 PMC7319177

[46] Chrysovergis K Wang X Kosak J Lee SH Kim JS Foley JF. NAG-1/GDF-15 Prevents Obesity by Increasing Thermogenesis, Lipolysis and Oxidative Metabolism. Int J Obes (Lond) (2014) 38(12):1555–64. doi: 10.1038/ijo.2014.27 PMC4135041 24531647

[47] Luan HH Wang A Hilliard BK Carvalho F Rosen CE Ahasic AM. GDF15 Is an Inflammation-Induced Central Mediator of Tissue Tolerance. Cell (2019) 178(5):1231–1244.e1211. doi: 10.1016/j.cell.2019.07.033 31402172 PMC6863354

[48] Suriben R Chen M Higbee J Oeffinger J Ventura R Li B. Antibody-Mediated Inhibition of GDF15–GFRAL Activity Reverses Cancer Cachexia in Mice. Nat Med (2020) . 26(8):1264–70. doi: 10.1038/s41591-020-0945-x 32661391

[49] Bosetti F. Arachidonic Acid Metabolism in Brain Physiology and Pathology: Lessons From Genetically Altered Mouse Models. J Neurochem (2007) 102(3):577–86. doi: 10.1111/j.1471-4159.2007.04558.x PMC2084377 17403135

[50] Hsu JY Crawley S Chen M Ayupova DA Lindhout DA Higbee J. Non-Homeostatic Body Weight Regulation Through a Brainstem-Restricted Receptor for GDF15. Nature (2017) 550(7675):255–9. doi: 10.1038/nature24042 28953886

[51] Chakraborty I Das SK Wang J Dey SK. Developmental Expression of the Cyclooxygenase-1 and Cyclo-Oxygenase-2 Genes in the Peri-Implantation Mouse Uterus and Their Differential Regulation by the Blastocyst and Ovarian Steroids. J Mol Endocrinol (1996) 16(2):107–22. doi: 10.1677/jme.0.0160107 9156514

