# Corrigendum: Arachidonic acid in follicular fluid of PCOS induces oxidative stress in a human ovarian granulosa tumor cell line (KGN) and upregulates GDF15 expression as a response

**DOI:** 10.3389/fendo.2022.988767

**Published:** 2022-10-04

**Authors:** Yalan Ma, Lianwen Zheng, Yeling Wang, Yiyin Gao, Ying Xu

**Affiliations:** ^1^ Reproductive Medical Center, Department of Obstetrics and Gynecology, The Second Hospital of Jilin University, Changchun, China; ^2^ Cardiovascular Medicine Department, The First Hospital of Jilin University, Changchun, China

**Keywords:** PCOS, arachidonic acid, oxidative stress (OS), human ovarian granulosa tumor cell line (KGN), GDF15, response

In the published article, there was an error. The product information of recombinant protein GDF15 that was present in the article needs to be corrected.

A correction has been made to **MATERIALS AND METHODS**, *Treatment of KGN Cells*, Paragraph Number 1. This sentence previously stated:

“KGN cells pretreated with or without buthionine sulfoximine (BSO) (10 mM)for 1h, and added with or without recombinant GDF15 (50ng/ml)(rGDF15; Abnova, Taipei City, Taiwan) for 4 h in advance, were also exposed to 50 mM AA for 12 h.”

The corrected sentence appears below:

“KGN cells pretreated with or without buthionine sulfoximine (BSO) (10 mM)for 1h, and added with or without recombinant GDF15 (50ng/ml)(rGDF15; Abnova, Taiwan, China) for 4 h in advance, were also exposed to 50 mM AA for 12 h.”

The authors apologize for this error and state that this does not change the scientific conclusions of the article in any way. The original article has been updated.

## Publisher’s note

All claims expressed in this article are solely those of the authors and do not necessarily represent those of their affiliated organizations, or those of the publisher, the editors and the reviewers. Any product that may be evaluated in this article, or claim that may be made by its manufacturer, is not guaranteed or endorsed by the publisher.

