# Supplementary material for: Arachidonic Acid in Follicular Fluid of PCOS Induces Oxidative Stress in a Human Ovarian Granulosa Tumor Cell Line (KGN) and Upregulates GDF15 Expression as a Response
Source: Front Endocrinol (Lausanne). 2022 May 11;13:865748. doi: 10.3389/fendo.2022.865748 (PMC9132262; doi:10.3389/fendo.2022.865748)
Supplement: Supplementary Table 1 — Comparison of fatty acid levels between the overweight PCOS patients 、the overweight control subjects、the normal PCOS subjects and the normal control subjects. [file DataSheet_1.docx]

**Supplementary Table 1 Comparison of fatty acid levels between the overweight PCOS patients 、the overweight control subjects、the normal PCOS subjects and the normal control subjects.**

| Fatty acids(（mg/kg） | Normal Control | Normal  PCOS | P-Value | the overweight control | the overweight  PCOS | P-Value |
| --- | --- | --- | --- | --- | --- | --- |
| C10:0 capric acid | 4.76±1.78 | 5.45±0.13 | 0.546 | 7.15±0.10 | 7.31±0.15 | 0.184 |
| C12:0 Lauric acid | 3.47±0.12 | 3.44±0.058 | 0.181 | 3.19±0.19 | 3.15±0.05 | 0.507 |
| C14:0 myristic acid | 6.61±0.13 | 6.84±0.11 | 0.07 | 7.21±0.06 | 7.19±0.04 | 0.789 |
| C15:0pentadecanoic acid | 2.64±0.16 | 2.68±0.16 | 0.73 | 3.50±0.05 | 3.35±0.095 | 0.077 |
| C16:0 palmitic acid | 276.3±1.52 | 273.6±3.55 | 0.294 | 138±1.0 | 275.33±0.577 | ＜0.01 |
| C16:1 palmitoleic acid | 6.50±0.49 | 7.26±0.28 | 0.081 | 3.74±0.13 | 6.16±0.07 | ＜0.01 |
| C17:0heptadecanoic acid | 2.34±0.12 | 2.23±0.11 | 0.336 | 2.91±0.12 | 3.13±0.11 | 0.092 |
| C18:0 stearic acid | 111±1.00 | 109±1.52 | 0.189 | 51.0±1.00 | 81.3±4.16 | ＜0.01 |
| C18:1n9c oleic acid | 109±1.00 | 107±2.00 | 0.196 | 71.6±1.52 | 131±1.00 | ＜0.01 |
| C18:2n6c linoleic acid | 123.3±7.6 | 109.3±1.52 | 0.081 | 111.17±0.76 | 239±1.00 | ＜0.01 |
| C18:3n3 linolenic acid | 4.21±0.08 | 4.06±0.06 | 0.058 | 6.05±0.63 | 6.32±1.17 | 0.518 |
| C20:1 cis-11-eicosanoic acid | 1.94±0.04 | 1.91±0.04 | 0.461 | 1.68±0.16 | 1.70±0.20 | 0.916 |
| C20:2 cis-11-eicosadienoic acid | 3.11±0.16 | 3.26±0.01 | 0.188 | 3.44±0.12 | 3.25±0.05 | 0.063 |
| C20:3n6cis-11-eicosatrienoic acid | 15.3±0.76 | 15.9±0.24 | 0.274 | 11.5±0.5 | 12.47±0.45 | 0.068 |
| C20:4n6 arachidonic acid | 28.00±0.24 | 37.05±0.35 | ＜0.01 | 38.00±3.0 | 33.78±0.68 | 0.076 |
| C20:5n3cis-5,8,111,14,17-eicosapentaenoic acid | 3.07±0.07 | 2.95±0.05 | 0.086 | 3.11±0.07 | 2.96±0.08 | 0.067 |
| C22.1n9 erucic acid | 74±3.6 | 69.16±1.15 | 0.091 | 56±1.00 | 55.3±5.03 | 0.833 |
| C22:6n3cis-4,7,10,13,16,19-Docosahexaenoic acid | 21.35±0.74 | 20±2.00 | 0.334 | 15.1±1.20 | 17.00±1.00 | 0.108 |
| SFA | 273.3±3.50 | 388.67±4.50 | ＜0.01 | 406.7±7.6 | 416.3±1.53 | 0.098 |
| MUFA | 208±8.02 | 197.3±7.50 | 0.158 | 120.3±2.08 | 204.7±4.04 | ＜0.01 |
| PUFA | 293.33±7.64 | 241.00±5.57 | ＜0.01 | 176.55±1.63 | 301.9±3.17 | ＜0.01 |

**Supplementary Table 2 Analysis of differences in gene expression between the two groups.**

| Gene ID | Gene name | Mean TPM  (AA) | Mean  TPM (NC) | log2FoldChange | P-Value | q-Value | result |
| --- | --- | --- | --- | --- | --- | --- | --- |
| ENSG00000204632 | HLA-G | 2.255881 | 0.407801666667 | 2.46775138942 | 0.000577020234385 | 0.0169923218489 | UP |
| ENSG00000147872 | PLIN2 | 58.8074763333 | 9.54791566667 | 2.62274185117 | 1.55791382794E-62 | 8.59449128414E-59 | UP |
| ENSG00000104419 | NDRG | 110.353075667 | 17.7374013333 | 2.63726027551 | 1.11988418528E-12 | 4.52050811374E-10 | UP |
| ENSG00000130513 | GDF15 | 300.249837333 | 29.027921 | 3.37065031037 | 0.00000743605526733 | 0.000588835955379 | UP |
| ENSG00000143333 | RGS16 | 0.821644666667 | 0.081381 | 3.33575069901 | 0.0000630979868286 | 0.0031934913823 | UP |
| ENSG00000109321 | AREG | 34.4412156667 | 8.08828633333 | 2.09023009015 | 6.37566672231E-18 | 7.5369488753E-15 | UP |
| ENSG00000153162 | BMP6 | 2.88831033333 | 0.482503333333 | 2.58161494607 | 0.0000000006557900089 | 0.000000172274994401 | UP |
